# Conservative Approach in Patients with Pemphigus Gingival Vulgaris: A Pilot Study of Five Cases

**DOI:** 10.1155/2014/747506

**Published:** 2014-11-23

**Authors:** Alessio Gambino, Mario Carbone, Paolo G. Arduino, Paola Carcieri, Lucio Carbone, Roberto Broccoletti

**Affiliations:** Department of Surgical Sciences, Oral Medicine Section, CIR Dental School, University of Turin, Via Nizza 230, 10126 Turin, Italy

## Abstract

*Objectives*. The aim of this pilot study was to describe the clinical efficacy of a conservative oral hygiene protocol in patients affected by gingival pemphigus vulgaris (PV) applied in a case series. *Methods*. Subjects suffering from PV with gingival localisation and slightly responsive to conventional treatment with systemic corticosteroids and immunosuppressive drugs were selected among individuals treated in the Unit of Oral Medicine Section of the University of Turin. Five subjects received nonsurgical periodontal therapy, over a 7-day period, including oral hygiene instructions; patients were instructed about domiciliary oral hygiene maintenance and instructions were reinforced at each visit and personalised if necessary. Clinical outcome variables were recorded at baseline (before starting) and 16 weeks after intervention, including full mouth plaque score (FMPS), bleeding scores (FMBS), probing pocket depth (PPD), oral pemphigus clinical score (OPCS), and patient related outcomes (visual analogue score of pain). *Results*. Five patients were treated and, after finishing the proposed therapy protocol, a statistical significant reduction was observed for FMBS (*P* = 0.043) and OPCS (*P* = 0.038). *Conclusions*. Professional oral hygiene procedures with nonsurgical therapy are related to an improvement of gingival status and a decrease of gingival bleeding in patients affected by PV with specific gingival localization.

## 1. Introduction

Pemphigus is a potentially life-threatening autoimmune mucocutaneous disease, usually characterised by blistering; pemphigus vulgaris (PV) is the main variant and the one that most commonly involves the mouth [1]. Gingival lesions are usual and may appear as unique blisters or erosions, predominantly on free gingiva, and may be challenging to diagnose as bullous lesions [2, 3].

The presence of epithelial desquamation, erythema, and erosive lesions on the gingival tissue is usually described as desquamative gingivitis (DG) [4]. It has been suggested that DG could play a role in increasing the long-term risk for periodontal tissue breakdown at specific sites [5]. We recently reported our experience in cases of DG: PV showed to be the less frequent pathology, representing 9.4% of all dysimmune diagnoses; however, when it affects the gingiva, lesions scarcely respond to immunosuppressive corticosteroid therapy [6]. Moreover, periodontal status has also been reported to be worse in PV patients, who should be encouraged to undergo long-term periodontal followup [7, 8].

The aim of this prospective case series was to evaluate the clinical efficacy of a professional oral hygiene protocol, followed by detailed oral hygiene instructions, in patients affected by PV with gingival localization.

## 2. Case Series Presentation

Subjects suffering from PV with gingival localisation (not completely responding to given systemic therapy) were selected among individuals attending the Unit of Oral Medicine Section of the University of Turin (Italy). The diagnosis was initially confirmed in all cases by histopathological examination and by direct immunofluorescence analysis.

Exclusion criteria included (I) current history of topical treatment for desquamative gingival lesions; (II) history of previous periodontal therapy (surgical and nonsurgical); (III) a number of teeth inferior to 18; (IV) pregnancy; and (V) diabetes mellitus. All eligible candidates for this study were informed about the experimental protocol and signed a consent form. The ethics review board of the Lingotto Dental School approved the study.

A prospective case series protocol (between January and December 2012), with nonsurgical periodontal therapy, was planned, similar to a previously reported one for patients with gingival lesions due to mucous membrane pemphigoid [9]. All individuals received a complete periodontal examination at baseline visit, including full mouth plaque scores (FMPS), full mouth bleeding upon probing scores (FMBS), probing pocket depth (PPD), and oral pemphigus clinical score (OPCS). OPCS is a parameter created for this study to value the presence or absence of erosions; it is performed on attributing to each sextant the value 1 for the presence of erosion and the value 0 for the absence of erosion.

Patient related outcomes included pain perception assessed at each visit by Visual Analogue Scale (VAS). The VAS consisted of a 10 cm horizontal line marked with 0 (= no pain) to 10 (= most severe pain experienced). Oral hygiene domiciliary scores (DOH) were recorded as detailed in Table 1. Followup visits were conducted 4 (T3) and 12 (T4) weeks after therapy. Clinical outcomes were subsequently reevaluated 16 weeks after the last treatment had been delivered (T5).

Subjects received nonsurgical periodontal therapy through supragingival scaling and polishing with the removal of all deposits and staining including oral hygiene instructions (Table 2). During each visit subjects were instructed about domiciliary oral hygiene maintenance; instructions were reinforced at each visit and personalised if necessary. Instructions included modified bass technique with soft brushes and a subsequent switch to medium brushes associated with interdental brushes. Patients were advised to change brushes every month and to change interdental brushes every 2 weeks [9]. Despite considering the fragility of the gingival tissues of these patients, no modifications were performed to reduce the trauma; surprisingly, tissues recovered extremely well immediately after the first week.

Comparative statistics were performed between T0 and T5. Paired samples test was used to test the difference in FMBS, FMPS, and PPD. Wilcoxon's signed rank was used to calculate the significance of the patient related outcomes (VAS and OPCS). *P* values ≤ 0.05 were considered to be statistically significant. SPSS (SPSS for windows, version 11, SPSS inc, Chicago, IL, USA) statistical software was utilized.

A total of 4 females and 1 male were recruited. The mean age at presentation was 59.5 years. All patients have been treated with systemic steroids (prednisone at 1–1.5 mg/kg in a single morning dose per o.s. at the beginning and then tapered if clinical and symptoms improvements were achieved) in the years before starting the protocol (medium treatment time of 102 months), still in partial remission with remaining gingival lesions.

A reduction in FMBS (*P* = 0.022) was observed after the clinical protocol was used. Moreover, a statistical significant reduction in patient's reported outcome was observed with a reduction in OPCS scores (*P* = 0.038) (Table 3).

**Figure 1:**
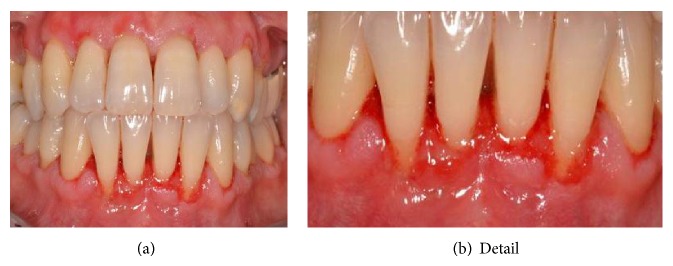
Clinical picture of the inferior frontal areas before periodontal treatment.

**Figure 2:**
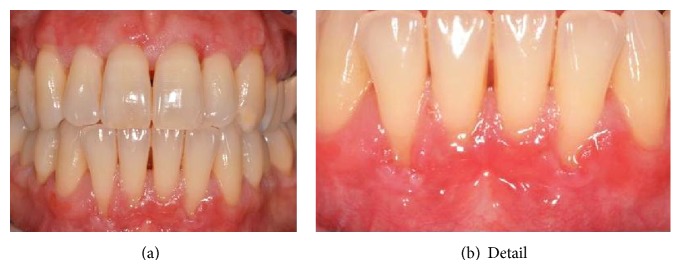
Clinical picture of the inferior frontal areas after periodontal treatment.

Despite the reduction of the FMPS and the PPD between T0 and T5, the analysis of these two parameters was not statistically significant (Figures 1 and 2).

## 3. Discussion

To the best of our knowledge, a case series of gingival PV patients, treated with conservative periodontal therapy, has never been reported.

Despite a lot of limitations, especially the small number of patients, our data suggests that oral hygiene therapy and professional instructions could be a successful mean to reduce clinical gingival inflammation and to improve patient related outcomes. The paucity of the reported sample is mainly due to the rarity of the disease and, moreover, to the infrequency on desquamative gingivitis not responding to the systemic therapy in PV patients.

The present study was developed by taking inspiration from the conclusions achieved by a previous work that underlined how professional oral hygiene procedures and nonsurgical periodontal therapy are connected with improvement of gingival status and decrease in gingival-related pain in patients affected by mucous membrane pemphigoid with specific gingival localization [9].

Patients affected by PV often experience pain and this leads to a greater discomfort when performing oral hygiene manoeuvres, mainly being afraid of causing new lesions and blisters [3]. Several authors had advocated the importance to maintain an optimal oral hygiene status in association to medical treatment in patients with PV with DG [1–4].

This work had the aim of evaluating whether the application of a protocol for causal therapy, coupled with precise instructions in oral hygiene in patients with gingival lesions by PV, may influence its clinical course. Bearing in mind this purpose, each PV patient underwent professional hygiene appointments. Without any earlier guidelines, the choice of starting with a soft toothbrush was based upon the notion of reducing the pain and discomfort; soon after an initial reduction of the gingival inflammation and as patients' confidence increased, each subject was advised to continue with a medium toothbrush [9]. We also investigated whether the causal therapy may decrease the signs and symptoms associated or, on the contrary, aggravate the autoimmune response, increasing erosion resulting from gingival epithelial blisters.

The analysis of the clinical parameters revealed a statistically significant reduction in FMBS and OPCS: this could be a sign of the reduction of gingival inflammation caused by bacterial deposits, which could positively influence the gingival response to autoimmune disease. Of course, the small size of the clinical sample does not allow any reliable nor conclusive data, but it certainly opens the way to a scientific hypothesis which could be potentially useful as part of the treatment plan of this rare disease.

Considering the small sample size we do not want to draw conclusions, but certainly the results reported could open future way of research. Moreover, further work with a much larger study population will be needed to verify the preliminary results obtained in this pilot study.

The oral hygiene protocol therefore represents a mean of primary, secondary, and tertiary prevention. PV patients need to be constantly followed by a team of specialists, amongst which we can mention oral hygienists and oral pathologists and all those who can diagnose and take care of pemphigus when it gives rise to manifestations in other districts (dermatologist, ophthalmologist, and otorhinolaryngologist).

However, based on our clinical preliminary results we could hypothesize that a protocol for causal therapy does not seem to aggravate the course of bullous-erosive lesions in patients with desquamative gingivitis caused by PV and reduces gingival bleeding. In addition to this, causal therapy seems to improve physical signs of autoimmune gingival disease, even though this does not lead to a significant change in the patient's symptoms.

## Figures and Tables

**Table 1:** The oral hygiene domiciliary scores^*^.

Score	Daily number of teeth brushing	Use of flossing device	Cleaning of the tongue
1	<1	No	No
2	1	No	No
3	1	Yes	Yes
4	≥2	Yes	Yes

^*^Taken from Arduino et al., 2011 [5].

**Table 2:** Clinical protocol used for gingival pemphigus vulgaris patients.

*Time 0 (T0)*: (i) Clinical evaluation and complete measurements^*^	

*Time 1_Day 07 (T1)*:	
(i) Scaling and prophylaxis of the upper sextants	
(ii) Oral hygiene instruction:	
(a) use a soft toothbrush^*α*^ for manual brushes, place the bristles at a 45° angle to the tooth surface at the gum edge, and then move the bristles back and forth in short (tooth-wide) strokes or small circular movements	
(iii) 0.20% chlorhexidine mouth rinse, for 1 minute,	
twice daily for 2 weeks	
(iv) VAS measurement	

*Time 2_Day 14 (T2)*:	
(i) Scaling and prophylaxis of the lower sextants	
(ii) VAS measurement	

*Time 3_Day 42 (T3)*:	
(i) Oral hygiene instruction:	
(a) use a medium toothbrush^*β*^ with a convenient handle	
(b) use a dental floss^*χ*^ for interdental plaque removal	
(ii) Clinical evaluation and VAS measurement	

*Time 4_Day 98 (T4)*:	
(i) Clinical evaluation and VAS measurements	

*Time 5_Day 126 (T4)*:	
(i) Clinical evaluation and complete measurements^*^	

^*^VAS (visual analogue scale) is a chromatic scale graduated from 0 (no pain) to 10 (unbearable pain), where it defines intensity of pain strong or very strong. The compilation of VAS is carried out at all appointments.

^*^FMBS (full mouth bleeding score) is performed on the first and last appointments.

^*^FMPS (full mouth plaque score) is performed on the first and last appointments.

^*^PPD (probing pocket depth) is performed on the first and last appointments.

^*^OPCS (oral pemphigus clinical score).

^*α*^Curasept soft CS 1560.

^*β*^Curasept medium CS 820.

^*χ*^Periofloss curaprox.

**Table 3:** The comparison of selected data at time 0 (T0) and at day 126 (T5).

	T0	T5	*P* ^*^
Full mouth bleeding score (%)	53.00 ± 15.05	27.80 ± 10.40	*0.043 *
Full mouth plaque score (%)	35.02 ± 15.23	26.20 ± 2.73	0.225
Probing depth	2.16 ± 0.45	2.12 ± 0.57	0.689
Referred symptoms (VAS score)	2.20 ± 1.79	1.60 ± 3.58	0.581
Oral hygiene domiciliary (DOH) scores	4.20 ± 0.44	4.40 ± 0.55	0.317
Oral pemphigus clinical score (OPCS) (%)	2.60 ± 2.07	1.20 ± 1.64	*0.038 *

^*^Test statistics: Wilcoxon signed-ranks test.
